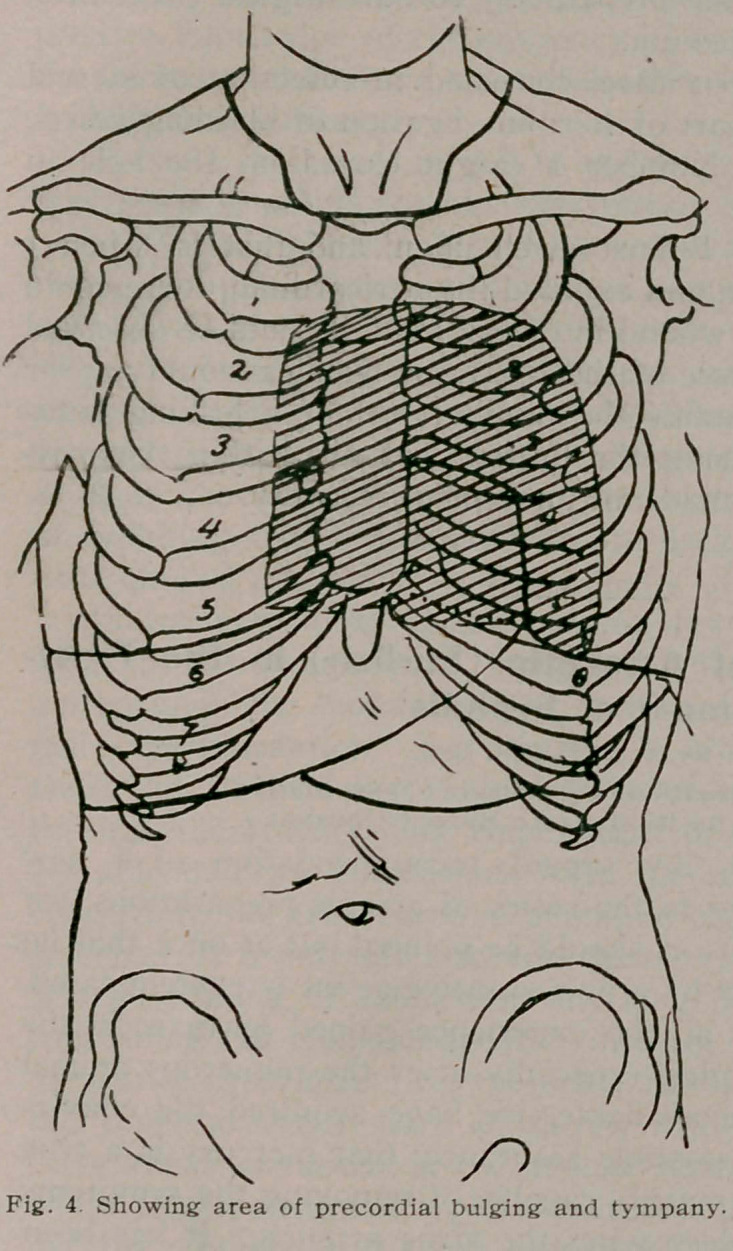# Pneumopericardium—With Report of a Case1Read at Buffalo Academy of Medicine, April, 13, 1909.

**Published:** 1909-05

**Authors:** Joseph Burke

**Affiliations:** Attending Surgeon, Emergency Hospital, Buffalo, N. Y.


### Pneumopericardium—with Report of a Case[1]

By JOSEPH BURKE, D. Sc., M. D.
Attending Surgeon, Emergency Hospital, Buffalo, N. Y.

THE presence of air in the pericardium is an occurrence of greatest rarity. Up to 1854, there were recorded but three authentic cases, those of Bricheteau, Stokes, and McDonald. Bamberger, as late as 1857, said that neither he nor Rokitansky (the greatest pathologist of his era), had ever seen a case of pneumopericardium. Schrœtter, another famous Viennese physician, in his thirty-five years of earnest and intelligent observation of thousands of cases in the Vienna General Hospital, affirmed that neither he nor his venerated teacher Skoda, had ever seen a case; hence, I feel that the extraordinary rarity alone of this pathological condition and the fact that I have a personal clinical case to report, will serve as my apology for its introduction and discussion here this evening.

1. Read at Buffalo Academy of Medicine, April, 13, 1909.

### ETIOLOGY.

The etiology of pneumopericardium is interesting; for, first, air can exist in the heart-sac spontaneously, as, for example, from the disintegration of a foul exudate, as in the case reported by Bricheteau; or, second, it can enter the pericardium from without the sac; (a) rupture of an air-containing abscess cavity, as in Graves's case of abscess of the liver which perforated the pylorus and pericardium; (b) communication of a tubercular vomica, or

Fig. 1. Percussion dulness, lying on back.

Fig. 2. Percussion dulness, sitting up.

of a pneumothorax, or ulcer of esophagus; (c) from external trauma, such as a stab wound and the like.

### SYMPTOMATOLOGY.

From the standpoint of symptomatology, the condition seems to have excited a great deal more attention in the early days of physical diagnosis than we find in the textbooks or medical journals of the present generation. I presume this lack of interest from the clinical point of view is due to the rarity of the affection. However, in many textbooks in the early 50's, especially in that of Stokes in 1855, we find portrayed most vividly the extraordinary physical signs that accompany it and are decidedly characteristic.

A painstaking review of the literature shows that in the cases reported, the physical phenomena were described so uniformly alike and were so remarkable, that in spite of the rare occurrence of the affection, its presence was very easily recognised. One must not forget however, that analogous to the behavior of pneumothorax, the pericardium can simultaneously contain not only air, but fluid as well, especially pus; therefore, one finds physical signs, according to whether fluid is also present or not. Classically, there is to be found bulging of the precordia, due to paresis of the intercostal muscles, remarkably shown in our own case and which first directed our attention; there is absence of the apex beat and cardiac impulse, when the patient is in the recumbent position which, however, reappears when he assumes the upright or lateral position, or bends the chest forward. Since the air in the pericardium occupies the upper portion of the sac, the law of gravity here obtains; the heart and exudate sink to the lower portion; the percussion phenomena become extraordinary. In the recumbent posture in place of the normal cardiac dulness, there appears a high pitched tympanitic note with accompanying metallic sound which, with diastole and systole, changes in character. As the patient changes from the horizontal to the upright position, the tympany gives place to normal cardiac dulness, and the apex beat reappears. In Walter James's case—I must say here that the clearest summary of the literature and most scientific exposition of the subject, pneumopericardium, was written by Dr. James in 1905—the study of the heart dulness in different positions of the body was

Fig. 3. Percussion dulness, lying on right side.

carefully observed and resulted as follows: "When the patient is lying on the back, there is pulmonary resonance with slightly tympanitic quality over the entire pericardium, as far down as the fifth costosternal junction, the line of dulness coming horizontally at this point. When the patient sits up, the percussion note from the third left space to the fifth rib becomes distinctly more dull; when lying on the right side, there is an area of dulness extending laterally from the right border of the sternum to a point half an inch to the left of the left border of the sternum, and longitudinally from the second interspace downward, until it merges into the liver dulness below."

I append herewith, figures 1, 2 and 3, illustrating Dr. James's case, and which are characteristic, taken from the *Medical Record,* June, 1905. The auscultatory phenomena are, according to Leube, more pregnant than percussion; the most important sign is the metallic character of the heart tones, which are so strong sometimes that they can be heard a distance away from the patient; if fluid be also present, there can be heard a peculiar succussion or splashing sound, which resembles the churning sound caused by a water wheel in motion.

In our own case the percussion findings were similar to those of Dr. Walter James's case, but the auscultation phenomena differed in this: that instead of succussion sounds there could be heard two dull, distant heart tones and a third dull, metallic sound, separated from these by a seeming interval; in other words, there could be heard three distinct tones, the first and second, dull and faint, the third characterised by a "metallic click;" there was no musical character to these sounds.

The functional signs that accompany pneumopericardium vary in different cases; in some there is dyspnea; in others, the difficulty in breathing is less. In our case there was neither cyanosis nor dyspnea; the patient acted as if there was nothing at all wrong with him.

DIFFERENTIAL DIAGNOSIS.

When one considers the above described individual physical signs of pneumopericardium, one cannot very easily confound this condition, if present, with any other; but, notwithstanding this, the following conditions may possibly be mistaken for it: dilated stomach, tubercular cavity and circumscribed pneumothorax. The one feature common to all of these conditions is the ability of the physician to determine the cardiac dulness and apex beat, when the patient is in the recumbent posture, readily determines the differential diagnosis.

J. F., male, 35 years, Italian. One Sunday, (April, 1908) about 4 P.M., while walking, patient was stabbed with a stilletto; he walked to his home several blocks away, and only on account of profuse bleeding, did he call a doctor three hours later. Unable to check the hemorrhage, which was becoming alarming, the doctor had patient removed to the Emergency Hospital in the ambulance. Upon examination, I found a small wound ¼ inch long at the junction of the second rib and sternum, from which profuse bleeding was taking place, which afterwards proved to be from the severed left internal mammary artery. Patient was in the easy recumbent posture; there was no cyanosis, no dyspnea though there was slight pallor. Inspection of chest revealed a very prominent, definitely circumscribed, bulging precordia, which corresponded to the classical area of relative cardiac dulness, increased one inch upward, one inch to right and left; there was, however, no perceptible apex beat, and no cardiac impulse could be felt.

Fig. 4. Showing area of precordial bulging and tympany.

(See Fig. 4). Percussion, patient recumbent, right chest. Normal resonance over whole lung anteriorly and posteriorly except from third rib downward, and from two fingers breadth, to right of right border of sternum, where the normal resonance was replaced by a peculiar metallic tympany, which differed from the pulmonary resonance; also from the gastric tympany in the same individual.

Percussion, left chest; normal resonance to second rib, then loud tympany downward, replacing the usual area of cardiac dulness in parasternal and mammillary lines; in auxillary line and posteriorly, normal resonance. When patient sits up and bends chest forward, the inspection and percussion findings change remarkably; the apex beat reappears, the cardiac impulse reasserts itself. I am sorry I did not examine patient in lateral positions. I cannot therefore, give findings.

I have already described the auscultatory findings in our case, in the discussion of the affection generally, so will not repeat them here. In view of these findings, we could not mistake the diagnosis which we made preparatory to our surgical procedure, that of air in the pericardium.

The treatment in our case, consisted in resection of sternal portion of second rib, part of sternum, ligation of bleeding vessel, retraction of pleura. Number 1 catgut closed up the hole in pericardium.

There is one point I must dwell upon, and that is, when I had retracted the pleura and exposed the pericardium, there could be seen a small ⅛ inch wound in the sac; and it could be observed that during every diastole of the heart, air bubbles would appear at the opening,—not unlike the familiar test of a leaking automobile tire-valve, showing the pressure of air within the pericardium. The patient made an uneventful recovery.

1092 Main Street.

## Figures and Tables

**Fig. 1. f1:**
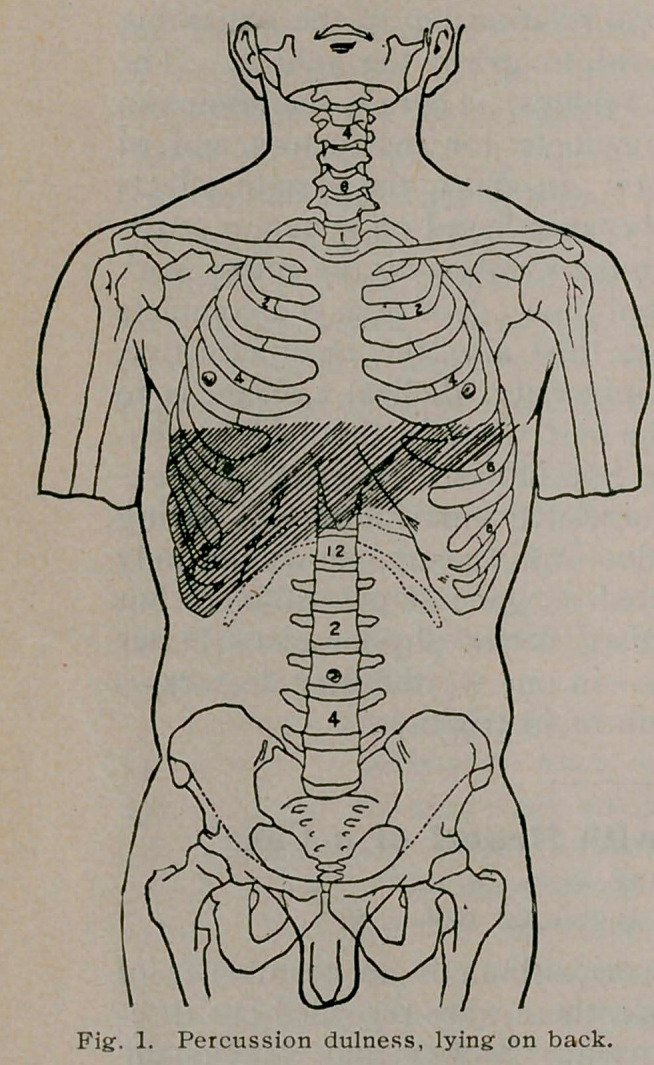


**Fig. 2. f2:**
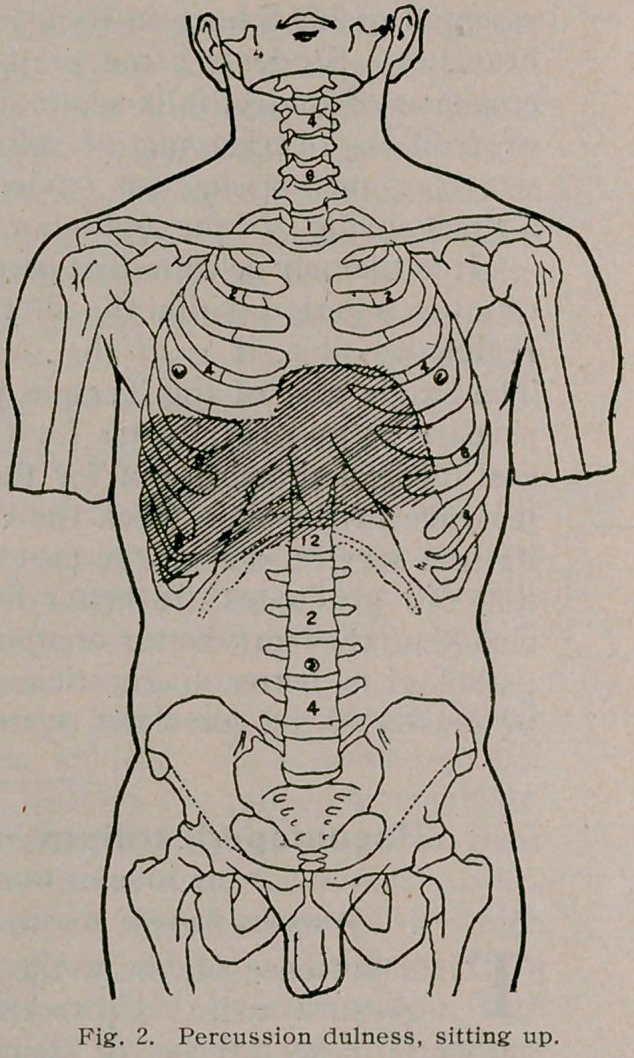


**Fig. 3. f3:**
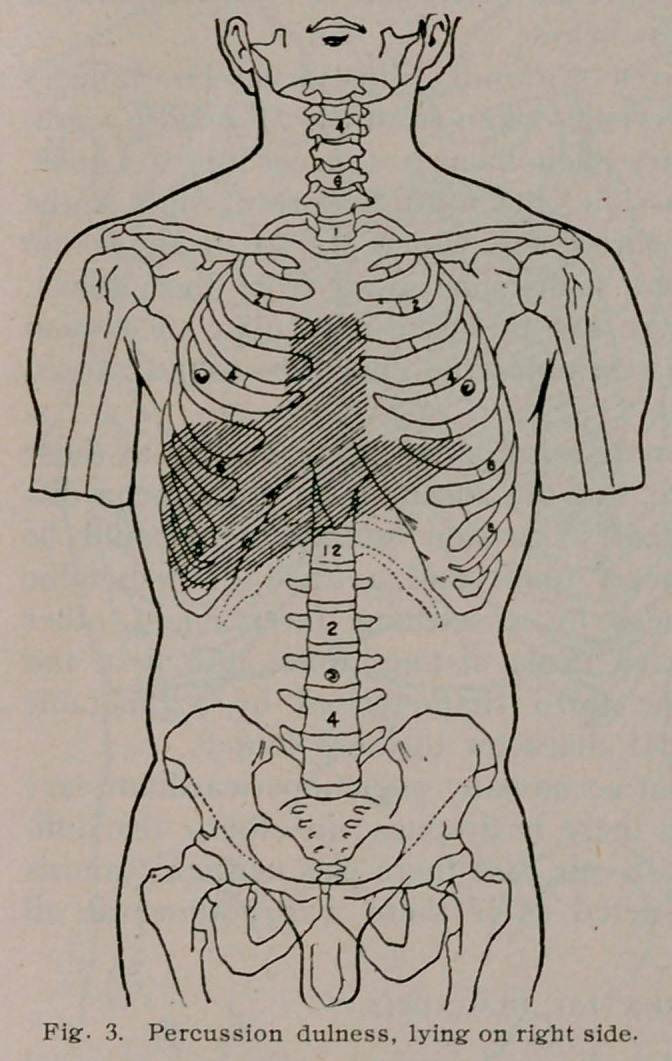


**Fig. 4. f4:**